# The management of chronic kidney disease in primary care in Denmark: patient characteristics, treatment, follow-up, progression and referral

**DOI:** 10.1093/ckj/sfae393

**Published:** 2024-11-30

**Authors:** Henrik Birn, Karl Emil Nelveg-Kristensen, Line Elmerdahl Frederiksen, Stefan Christensen, Juha Mehtälä, Sarah Smith, Michael Bruun, Ulrik Bodholdt

**Affiliations:** Department of Renal Medicine, Aarhus University Hospital, Department of Clinical Medicine and Biomedicine, Aarhus University, Aarhus, Denmark; Department of Nephrology, Rigshospitalet, Copenhagen University Hospital, Copenhagen, Denmark; Medical Evidence, Medical & Regulatory, Biopharmaceuticals, AstraZeneca, Copenhagen, Denmark; Cardiovascular, Renal and Metabolism, Medical Affairs Department, Biopharmaceuticals, AstraZeneca, Copenhagen, Denmark; MedEngine Oy, Helsinki, Finland; MedEngine Oy, Helsinki, Finland; Lægerne Ved Fjorden, Struer, Denmark; Kastruplægerne, Copenhagen, Denmark

**Keywords:** chronic kidney disease, clinical characteristics, disease management, primary health care

## Abstract

**Background:**

Chronic kidney disease (CKD) is mainly managed in primary care, but detailed information on these patients is limited. This study describes CKD patients and the disease management and referrals by general practitioners (GPs) in Denmark in order to identify opportunities for improved care.

**Methods:**

Patients with CKD, defined by at least two abnormal estimated glomerular filtration rate (eGFR) or urinary albumin/creatinine ratio (UACR) measurements ≥90 days apart during 2019–2020, were followed until May 2023 utilizing electronic health records.

**Results:**

Among 1316 patients with one abnormal eGFR or UACR test, 993 (75%) had a second abnormal test within a median of 10.8 months, which confirmed CKD. Most patients (62%) were G-stage 3a, 89% had cardiovascular disease and 34% had diabetes. A UACR test was performed in 52% of patients around time of index. The use of renin–angiotensin–aldosterone system inhibitors was high (67%), whereas sodium-glucose cotransporter 2 inhibitors was low at inclusion (5%), although increasing during follow-up (15%). Patients had a median of 13.5 GP contacts/year, 1–2 eGFR and 0–1 UACR tests/year, and only 2.7% were referred to a nephrologist. The median decline in eGFR was modest; however, 15% experienced a drop of >5.0 mL/min/1.73 m^2^ during 3-years of follow-up.

**Conclusions:**

The findings indicate a high likelihood of CKD following one abnormal measurement. CKD patients constitute a significant burden to primary care with frequent GP contacts, yet more focus on UACR testing and new treatment adaptation to improve CKD prognosis is warranted.

## Graphical Abstract

**Graphical Abstract:**
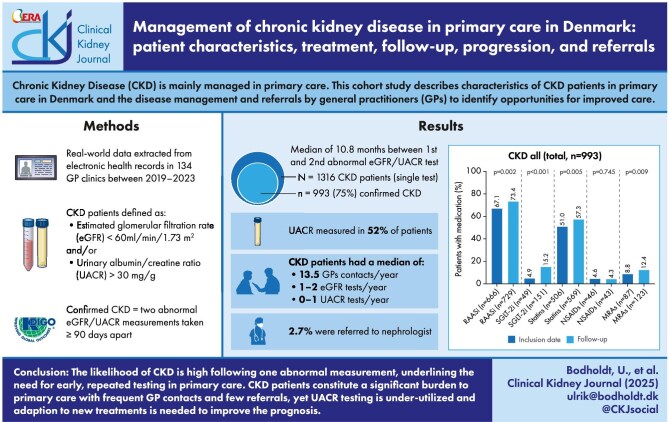

KEY LEARNING POINTS **What was known:**

- Chronic kidney disease (CKD) affects approximately one in 10 adults globally, is associated with high morbidity and mortality, and yet, is underdiagnosed and undertreated.
- Most CKD patients are treated in primary care and barriers to optimal detection and management of CKD may still exist.
- To optimize strategies aiming to advance CKD management in primary care, more knowledge is required in order to identify potential inadequacies.

**This study adds:**

- Most patients with a single marker of declined kidney function had their CKD confirmed by a second abnormal estimated glomerular filtration rate or urinary albumin/creatinine ratio (UACR) measurement with a median time of 10.8 months.
- UACR remains widely underutilized in primary care.
- The CKD patient population is a substantial resource burden to primary care with frequent general practitioner contacts and low referral rates to nephrologists.

**Potential impact:**

- This study points to the need for increased utilization of readily available UACR and timely confirmation of CKD in primary care.
- The results indicate that the use of recommended treatments to decrease CKD progression, including optimal blood pressure control and sodium-glucose cotransporter 2 inhibitors, are not fully implemented, although they are increasingly applied.
- The study points to the need for relevant tools to identify CKD patients at risk of rapid progression to allow the allocation of resources and follow-up to those at greatest risk.

## INTRODUCTION

Chronic kidney disease (CKD) is a potentially progressive and life-threatening condition that presents a major global health challenge [1, 2]. Approximately 10% of the adult population has CKD [2, 3]. In most cases, CKD can be identified by repeated measurements of estimated glomerular filtration rate (eGFR) and urinary albumin/creatinine ratio (UACR). Furthermore, CKD can be classified into stages defining the associated risks using the KDIGO recommendations [4].

Despite the relative simplicity of diagnosing CKD and the availability of guideline-directed therapies reducing the risk of progression and mortality, CKD remains underdiagnosed and undertreated [5–8]. A recent multi-country study indicated that two out of three patients fulfilling the laboratory criteria of CKD did not have a CKD-specific diagnostic code [3].

In many countries including Denmark, mild and moderate CKD is diagnosed and managed in primary care [9, 10]. Thus, strategies identifying patients and improving management in primary care are crucial for reducing the incidence of severe CKD, and associated comorbidities and mortality [9]. In Denmark, healthcare is tax-funded with free, universal access to both primary and specialized care [11]. However, barriers to optimal CKD detection and management may still exist [12]. These include the lack of symptoms associated with early CKD stages, lack of patient and physician awareness, lack of a widely used diagnostic code for CKD in primary care in Denmark, and insufficient knowledge or resources allocated to clinical evaluation and treatment of CKD and associated risks [10, 12, 13]. In addition, inadequate follow-up and risk assessment possibly lead to a lack of or late referral to specialized care [10, 14]. In Denmark, approximately 700 new cases of end-stage kidney failure are diagnosed annually [15], highlighting the need for earlier detection to mitigate kidney disease progression. For designing strategies to optimal CKD management in primary care, a better country-specific understanding is needed to identify potential inadequacies and barriers to improved care. Existing literature on quality-of-care assessment of CKD management in primary care includes studies from the Netherlands [16], the USA [17] and Canada [18], but with large methodological heterogeneity in definition of CKD and quality indicator assessments. Moreover, to identify both healthcare system–related and demographic barriers to improved CKD care management, an understanding of the local healthcare context is paramount. Borg *et al*. [19] reported on CKD management in Denmark, utilizing a large primary care cohort of CKD patients identified between 2001 and 2015, but mainly reported on the association between eGFR and clinical outcomes, such as cardiovascular disease and mortality. Thus, we believe that the current study adds additional and updated real-world data on the diagnosis and management of CKD in the primary care setting in Denmark.

This study aimed to address the evidence gaps on CKD management of general practitioners (GPs) in Denmark by identifying CKD patients in primary care and describing their characteristics, treatment, primary healthcare utilization, CKD progression and referral to nephrology care.

## MATERIALS AND METHODS

### Design

This national cohort study utilized data collected retrospectively from electronic healthcare records (EHRs) in primary care. Selected GP clinics representing all five geographical regions of Denmark were invited to participate between April and October 2023. During or after data extraction by the GP, no personal identification number of patients was revealed to the clinical research associate (CRA) or stored anywhere, and data was statistically processed on an aggregated level.

### Eligibility screening for CKD patients

Eligible patients were identified from EHRs of the participating GP clinics. Since no specific, widely used diagnostic code for CKD exist in primary care, step-by-step search manuals were developed enabling the GPs to perform a retrospective, digital screen in their EHR system (XMO, NOVAX, WinPLC and EG Clinea), and identify possible CKD patients [20]. Patients were eligible for inclusion when having at least one laboratory measurement of eGFR <60 mL/min/1.73 m^2^ and/or UACR >30 mg/g during the inclusion period (1 January 2019 to 31 December 2020), and age ≥18 years. The date of the first eligible eGFR or UACR served as study inclusion date. From all possible CKD patients, a maximum of 10 eligible patients were randomly selected from each GP clinic.

Following the random selection of patients, the presence of any exclusion criteria at inclusion date was checked individually by the GP and a trained CRA. Exclusion criteria were pregnancy, terminal illness (i.e. patients requiring mostly palliative care), active chemotherapy treatment, a history of chronic dialysis (>3 months), kidney transplantation or previous referral to a nephrologist. The latter was applied to avoid exclude prevalent CKD patients followed by renal specialists in secondary care setting and therefore not representative when exploring CKD care in the GP setting.

### Study population

The study population comprises patients with confirmed CKD defined by a second confirmatory measurement of eGFR <60 mL/min/1.73 m^2^ and/or UACR >30 mg/g at least 90 days after their first eligible eGFR and/or UACR measurement at study inclusion date. The tests were required to be of the same type (i.e. two eGFR or UACR tests). The date of the confirmatory measurement was defined as the index date.

### CKD staging

CKD G-stage was defined by the second confirmatory eGFR measurement for patients who were included in the study based on eGFR measurement. For patients included by UACR measurement, CKD G-stage was determined by the first available eGFR measurement at any time since inclusion. If no eGFR measurement was available (i.e. only UACR measurements), patients were not allocated to a CKD stage but remained included in the total study population (Supplementary data, Table S1).

### Follow-up period

The follow-up period started at the date of the second confirmatory measurement (i.e. index) and continued until the time of death, change of GP, emigration, referral to a nephrologist or end of the study period (31 May 2023), whichever occurred first.

### Data collection

Data collection was performed during June–November 2023 by two CRAs in close collaboration with the GPs. Each participating GP (or another healthcare professional representative from the GP clinic) performed a retrospective manual search in the EHRs of eligible patients. The pre-specified patient information was orally passed to the CRA who entered the anonymized data into a standardized electronic case report form (MyCRF).

Sociodemographic characteristics (age, sex, smoking status), comorbidities [diabetes, cardiovascular disease (CVD) and urinary tract infections], and CKD-related baseline information were collected at the inclusion date. All eGFR and UACR measurements, the total number of GP consultations and referrals to a nephrologist were collected across the entire follow-up period. Other laboratory measurements [hemoglobin (Hb), hemoglobin A1c (HbA1c) and low-density lipoprotein (LDL)], blood pressure (BP) and active prescriptions of CKD-relevant medication groups [sodium-glucose cotransporter 2 inhibitors (SGLT-2i), renin–angiotensin–aldosterone system inhibitors (RAASi), statins, nonsteroidal anti-inflammatory drugs (NSAIDs), mineralocorticoid receptor antagonists (MRAs)] were collected from the closest measurement (±4 weeks) from the study inclusion date and the latest available measurement before the end of follow-up.

Laboratory values were considered abnormal if Hb was <8.3 mmol/L for men or <7.3 mmol/L for women, HbA1c >48 mmol/mol or LDL >3.0 mmol/L, while BP was considered high if systolic BP >130 mmHg and/or diastolic BP >85 mmHg.

To estimate GFR, the Chronic Kidney Disease Epidemiology Collaboration 2009 equation without correction for race is used in primary care in Denmark [21].

### Statistical analyses

Data were presented using descriptive statistics; for non-normally distributed data, median with interquartile range (IQR) was used. All analyses were stratified by CKD stage at index. Results were masked for data fields representing fewer than five individuals due to data sensitivity. Significant differences in medication use were determined using the Chi-square test. A *P*-value of <.05 was considered statistically significant. To assess eGFR decline over time, a linear model was fitted separately for each individual, using all available eGFR values from index date up to 3 years after index, and considering both decreasing and increasing eGFR values. The estimated slope from the linear model was summarized as median values. Analyses were conducted using the statistical software R (version 4.0.3 or higher, http://www.r-project.org).

## RESULTS

### Formation of the study population

**Figure 1:**
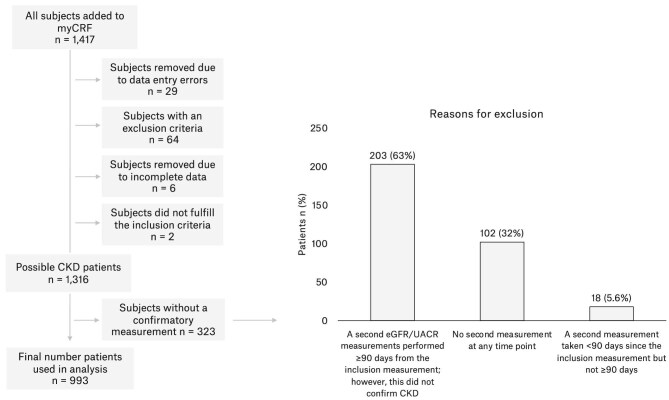
Formation of the study population. A total of 134 GP clinics across Denmark accepted the invitation to participate. The EHR search from each contributing GP identified, on average, 191 patients per site with possible CKD fulfilling the initial single test screening criteria. Left: formation of the study population from the 1417 patients added to MyCRF. Right: reasons why 323 possible CKD patients did not fulfill the criteria for confirmed CKD. Of the 323 patients, 175 patients had been included as possible CKD patients by eGFR measurement and 151 by UACR measurement (3 patients fulfilled both criteria). MyCFR, electronic case report form.

A total of 134 GP clinics participated, from which 1316 randomly selected patients were included based on a single laboratory measurement suggesting impaired kidney function (Fig. 1). Among the 1316 patients, 1196 (91%) had a second laboratory measurement performed ≥90 days after, and 993 (75%) patients were included in the study population of confirmed CKD patients based on their second, confirmatory measurement of declining kidney function test result ≥90 days after the initial test. The median time from the first inclusion measurement to the confirmatory measurement was 10.8 (IQR 6.0–18.0) months. Of the CKD patients, most (*n* = 776, 78%) were included based on an eGFR test, while 217 (22%) were included based on a UACR test.

### Patient characteristics

The majority (*n* = 723, 73%) of patients were ≥70 years and 534 (54%) were females (Table 1). Most patients (*n* = 613, 62%) were classified as CKD G-stage 3a at index. Half of the study population (*n* = 520, 52%) had a UACR test performed within 7 days after the index date. Among these patients, 207 (40%) had UACR <30 mg/g, 273 (53%) had UACR 30–300 mg/g and 40 (8%) had UACR >300 mg/g. Within the group of patients with CKD stage G4, 74% did not have a UACR test performed within 7 days after the index date.

**Table 1:** Baseline sociodemographic and clinical characteristics of confirmed CKD patients, stratified by the CKD stage.

	CKD stage at index (*n*, patients)					
	Total (*N* = 993, 100.0%)^a^	G1 (*n* = 104, 10.5%)	G2 (*n* = 103, 10.4%)	G3a (*n* = 613, 61.8%)	G3b (*n* = 149, 15.0%)	G4 (*n* = 23, 2.3%)
Sociodemographic characteristics						
Gender, *n* (%)						
Female	534 (53.8)	45 (43.3)	39 (37.9)	352 (57.4)	87 (58.4)	11 (47.8)
Male	459 (46.2)	59 (56.7)	64 (62.1)	261 (42.6)	62 (41.6)	12 (52.2)
Age group, *n* (%)						
<59 years	101 (10.2)	49 (47.1)	12 (11.7)	35 (5.7)	<5	<5
60–69 years	169 (17.0)	31 (29.8)	31 (30.1)	95 (15.5)	11 (7.4)	<5
70–79 years	393 (39.6)	21 (20.2)	38 (36.9)	275 (44.9)	54 (36.2)	5 (21.7)
80 years or over	330 (33.2)	<5	22 (21.4)	208 (33.9)	81 (54.4)	16 (69.6)
Smoking, *n* (%)						
Current smoker	148 (14.9)	27 (26.0)	17 (16.5)	84 (13.7)	18 (12.1)	<5
Former smoker	258 (26.0)	24 (23.1)	36 (35.0)	159 (25.9)	34 (22.8)	5 (21.7)
Never smoked	461 (46.4)	43 (41.3)	43 (41.7)	297 (48.5)	69 (46.3)	9 (39.1)
Missing	126 (12.7)	10 (9.6)	7 (6.8)	73 (11.9)	28 (18.8)	8 (34.8)
CKD-related information						
UACR at index (measured within 7 days after index date), *n* (%)						
A1 (<30 mg/g)	207 (39.8)	0 (0.0)	0 (0.0)	174 (67.4)	31 (64.6)	<5
A2 (30–300 mg/g)	273 (52.5)	90 (86.5)	88 (85.4)	77 (29.8)	13 (27.1)	<5
A3 (>300 mg/g)	40 (7.7)	14 (13.5)	15 (14.6)	7 (2.7)	<5	0 (0.0)
Missing	473 (47.6)	0 (0.0)	0 (0.0)	355 (57.9)	101 (67.8)	17 (73.9)
Cause of CKD, *n* (%)						
Diabetic kidney disease	223 (22.5)	63 (60.6)	36 (35.0)	88 (14.4)	29 (19.5)	6 (26.1)
Hypertensive nephropathy	285 (28.7)	25 (24.0)	31 (30.1)	181 (29.5)	40 (26.8)	7 (30.4)
Atherosclerosis of renal artery	41 (4.1)	<5	<5	24 (3.9)	12 (8.1)	<5
Obstructive nephropathy	32 (3.2)	<5	<5	18 (2.9)	5 (3.4)	<5
Unknown^b^	504 (50.8)	25 (24.0)	44 (42.7)	348 (56.8)	77 (51.7)	10 (43.5)
Other previous renal-related diseases or operations, *n* (%)						
No^b^	930 (93.7)	102 (98.1)	98 (95.1)	579 (94.5)	132 (88.6)	18 (78.3)
Acute renal failure	7 (0.7)	0 (0.0)	0 (0.0)	6 (1.0)	<5	0 (0.0)
Renal cancer	9 (0.9)	0 (0.0)	<5	5 (0.8)	<5	0 (0.0)
Surgery on kidney or bladder	21 (2.1)	<5	<5	10 (1.6)	6 (4.0)	3 (13.0)
Other	33 (3.3)	<5	<5	17 (2.8)	9 (6.0)	<5
Comorbidities at time of study inclusion						
Diabetes at inclusion, *n* (%)						
Type 1 diabetes	15 (1.5)	<5	<5	7 (1.1)	<5	0 (0.0)
Type 2 diabetes	336 (33.8)	71 (68.3)	50 (48.5)	165 (26.9)	41 (27.5)	8 (34.8)
Cardiovascular disease at inclusion, *n* (%)						
Hypertension	734 (73.9)	76 (73.1)	86 (83.5)	444 (72.4)	110 (73.8)	17 (73.9)
Ischemic heart disease	140 (14.1)	7 (6.7)	14 (13.6)	90 (14.7)	24 (16.1)	5 (21.7)
Heart failure	85 (8.6)	<5	<5	47 (7.7)	27 (18.1)	<5
Stroke	72 (7.3)	<5	7 (6.8)	41 (6.7)	20 (13.4)	<5
Peripheral vascular disease	44 (4.4)	6 (5.8)	<5	25 (4.1)	10 (6.7)	<5
Other cardiovascular diseases	410 (41.3)	42 (40.4)	37 (35.9)	261 (42.6)	58 (38.9)	12 (52.2)
Other cardiovascular diseases at inclusion, *n* (%)						
Diabetic circulatory complications	44 (4.4)	16 (15.4)	5 (4.9)	16 (2.6)	5 (3.4)	<5
Peripheral artery diseases	144 (14.5)	15 (14.4)	14 (13.6)	97 (15.8)	13 (8.7)	5 (21.7)
Cardiac valve disorders	71 (7.2)	5 (4.8)	5 (4.9)	54 (8.8)	7 (4.7)	0 (0.0)
Atrial fibrillation	176 (17.7)	6 (5.8)	14 (13.6)	116 (18.9)	33 (22.1)	7 (30.4)
Cerebrovascular disease excluding stroke, other occlusions of cerebral arteries and transient cerebral ischemic attack	37 (3.7)	<5	6 (5.8)	24 (3.9)	<5	<5
Cardiac arrest	<5	0 (0.0)	0 (0.0)	0 (0.0)	<5	0 (0.0)
Urinary tract infection at inclusion, *n* (%)						
Yes	43 (4.3)	7 (6.7)	<5	19 (3.1)	11 (7.4)	<5

a Patients with unknown CKD stage are not reported separately but are included in the “total” column due to a low number of observations (<5).

b Derived variable: No other options were collected to electronic case report form.

<5 = variables for which a low number (<5) of observations prevent tabulation of the exact counts due to data sensitivity.

Q1, first quartile; Q3, third quartile.

Patient and clinical characteristics stratified by diabetes are presented in Supplementary data, Table S2. Of patients with diabetes, 225 (64%) had a UACR test performed within 7 days after the index date. The median follow-up time was 2.6 (IQR 1.7–3.3) years.

### Comorbidities

Most patients (*n* = 880, 89%) were diagnosed with CVD, most commonly hypertension (*n* = 734, 74%) or atrial fibrillation (*n* = 176, 18%) (Table 1). Heart failure was present in 85 (9%) patients, being more than twice as prevalent in stage G3b patients compared with G3a. Approximately one in three (*n* = 336, 34%) patients had type 2 diabetes. The proportion of type 2 diabetes patients was highest in stages G1 (*n* = 71, 68%) and G2 (*n* = 50, 49%). When stratified by patients with a single eligible eGFR or UACR test and patients with confirmatory testing, respectively, we observed that patients with a single test were younger and less likely to have diabetes (24% vs 34%), hypertension (48% vs 74%) and other cardiovascular diseases (25% vs 41%) than confirmed CKD patients (Supplementary data, Table S3).

### Laboratory measurements

**Figure 2:**
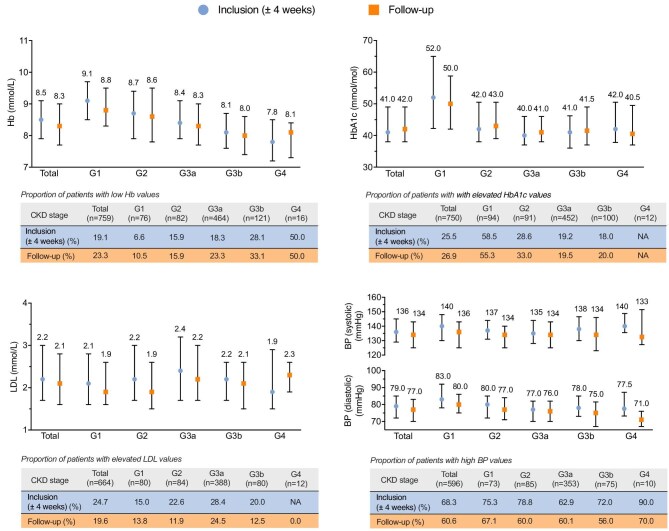
Hb, HbA1c, BP and LDL values of the CKD patients, total and by CKD G-stage at inclusion. Median values at the time of inclusion (closest measurement during ±4 weeks from the inclusion date) and the end of follow-up (the closest measurement) and the proportion of patients with low Hb (<8.3 mmol/L for men and <7.3 mmol/L for women); elevated HbA1c (>48 mmol/mol); elevated LDL (>3.0 mmol/L); or high BP (>130/85 mmHg, defined by either increased systolic or diastolic value) and stratified by the CKD stage at index. Percentages in brackets after the CKD stage indicate the proportion of individuals with a measurement available. NA = not available; these subgroups included <5 patients and thus they are masked.

The median values of the laboratory measurements and BP of patients who had measurements available both at the time of inclusion and the end of follow-up are shown in Fig. 2. At the time of inclusion, high BP was observed in 407 (68%) patients, elevated HbA1c in 191 (26%), elevated LDL in 159 (25%) and low hemoglobin in 145 (19%) patients. At the end of follow-up, a slightly higher proportion of patients had elevated HbA1c (*n* = 202, 27%) and low hemoglobin (*n* = 177, 23%), and a slightly lower proportion had high BP (*n* = 361, 61%) and elevated LDL (*n* = 126, 20%). Only minor changes in median values of laboratory and BP measurements were observed during the follow-up (Table 2).

**Table 2:** Changes in the median values of different laboratory and BP measurements between the time of inclusion and end of follow-up in patients with both measurements available.

	Stratification by CKD G-stage at index (*N*, patients)					
	Total (*N* = 993, 100.0%)^a^	G1 (*n* = 104, 10.5%)	G2 (*n* = 103, 10.4%)	G3a (*n* = 613, 61.8%)	G3b (*n* = 149, 15.0%)	G4 (*n* = 23, 2.3%)
Hb measurement available, *n* (%)	759 (76.4)	76 (73.1)	82 (79.6)	464 (75.7)	121 (81.2)	16 (69.6)
Hb (mmol/L), median (Q1, Q3)	–0.1 (–0.5, 0.3)	–0.1 (–0.7, 0.3)	–0.1 (–0.4, 0.3)	–0.1 (–0.5, 0.3)	0.0 (–0.5, 0.4)	0.0 (–0.4, 0.3)
HbA1c measurement available, *n* (%)	750 (75.5)	94 (90.4)	91 (88.3)	452 (73.7)	100 (67.1)	12 (52.2)
HbA1c (mmol/mol), median (Q1, Q3)	–0.2 (10.5)	–5.4 (17.0)	0.0 (9.6)	0.5 (9.0)	1.2 (6.7)	2.3 (8.0)
LDL measurement available, *n* (%)	644 (64.9)	80 (76.9)	84 (81.6)	388 (63.3)	80 (53.7)	12 (52.2)
LDL (mmol/L), median (Q1, Q3)	–0.1 (–0.5, 0.3)	–0.1 (–0.5, 0.3)	–0.1 (–0.6, 0.3)	–0.0 (–0.4, 0.3)	–0.1 (–0.4, 0.2)	–0.1 (–0.3, 0.5)
Systolic BP measurement available, *n* (%)	596 (60.0)	73 (70.2)	85 (82.5)	353 (57.6)	75 (50.3)	10 (43.5)
Systolic BP (mmHg), median (Q1, Q3)	–2.0 (–15.0, 9.0)	–5.0 (–16.0, 8.0)	–5.0 (–15.0, 5.0)	–1.0 (–14.0, 9.0)	–2.0 (–15.0, 10.0)	–11.5 (–15.8, 16.0)
Diastolic BP measurement available, *n* (%)	595 (59.9)	73 (70.2)	85 (82.5)	352 (57.4)	75 (50.3)	10 (43.5)
Diastolic BP (mmHg), median (Q1, Q3)	–1.0 (–9.0, 5.0)	–3.0 (–11.0, 3.0)	–1.0 (–9.0, 4.0)	–1.0 (–8.2, 6.0)	–3.0 (–11.5, 3.5)	–6.0 (–18.8, 1.5)

a Patients with unknown CKD stage are not reported separately but are included in the “total” column due to a low number of observations (<5).

### Medication use

**Figure 3:**
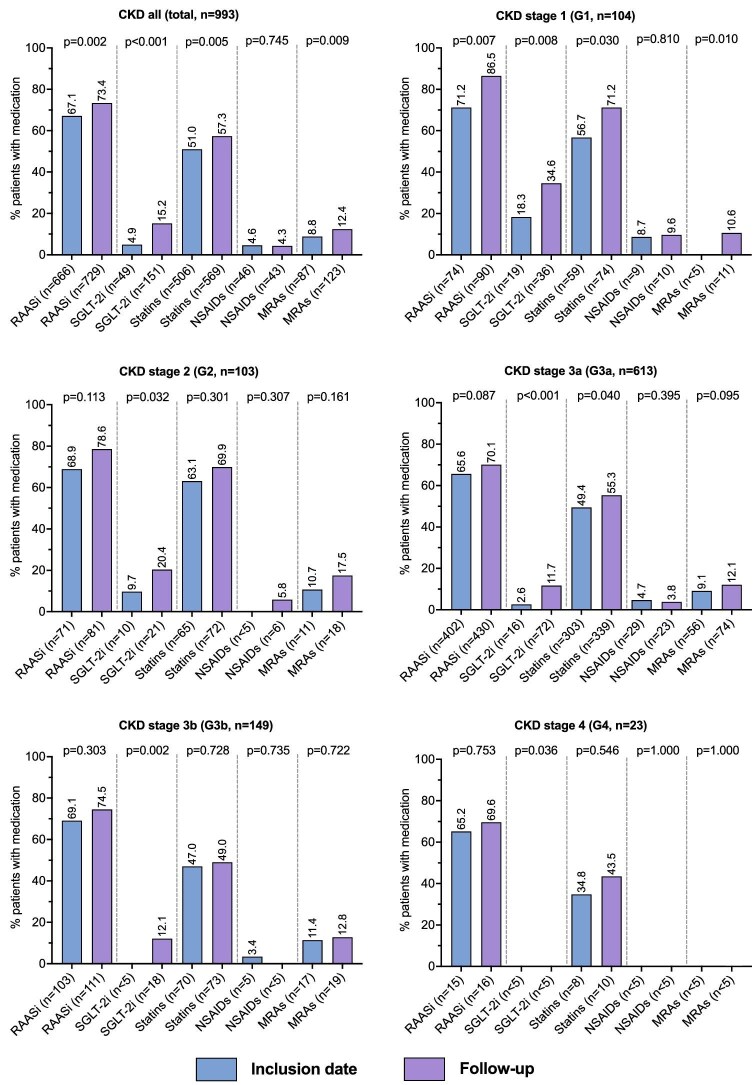
Medication prescriptions in the study population, total and by CKD G-stage at inclusion. Comparison of the proportion of patients in the study population with an active prescription of different medication groups at the inclusion date and the end of follow-up. RAASi (ATC group: C09); SGLT-2i (ATC group: A10BK); statin (ATC group: C10AA); MRA (ATC group: N04A).

At the inclusion date, RAASi were prescribed to 666 (67%), statins to 506 (*n* = 51%), MRAs to 87 (9%), SGLT-2i to 49 (5%) and NSAIDs to 46 (5%) of the patients (Fig. 3). All medications, except NSAIDs were prescribed to a higher proportion of patients at the end of follow-up compared with the inclusion date. The largest increase was observed for SGLT-2i (*n* = 49, 5%, to *n* = 151, 15%, *P* < .001). At the end of follow-up, SGLT-2i were most commonly prescribed in stages G1 (*n* = 36, 35%) and G2 (*n* = 21, 20%) and more often to patients with diabetes compared with non-diabetics (*n* = 132/351, 38% to *n* = 19/642, 3%) (Supplementary data, Table S2). In addition, the use of MRAs was more common in diabetics compared with non-diabetics. Patients with a single eligible eGFR or UACR test were less likely to have RAASi prescriptions (47% vs 67%) and annual GP consultations (10 vs 13.5) than confirmed CKD patients (Supplementary data, Table S3).

### Annual eGFR and UACR measurements during follow-up

**Figure 4:**
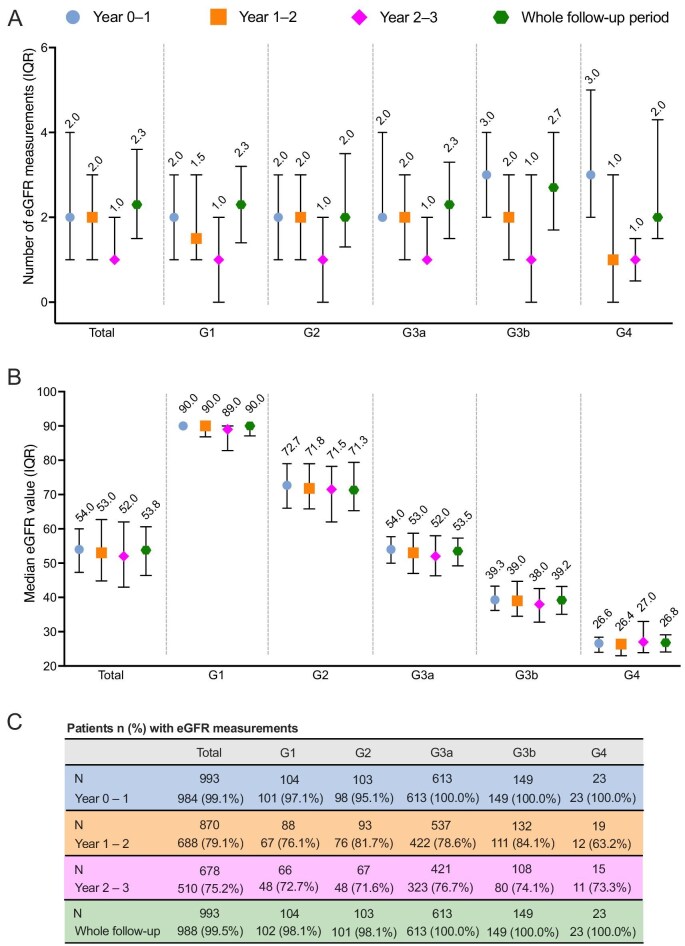
Median number and values of eGFR measurements during 3 years of follow-up in the study population, total and by CKD G-stage at index. (**A**) Median number of eGFR measurements, (**B**) median eGFR values and (**C**) number and fraction of patients with eGFR measurements. All measurements taken during a given year were included in the calculation of the number and values of eGFR measurements for that year. For the whole follow-up period analyses, all measurements during the follow-up were included and the annual number of measurements was calculated by dividing by the length of follow-up.

**Figure 5:**
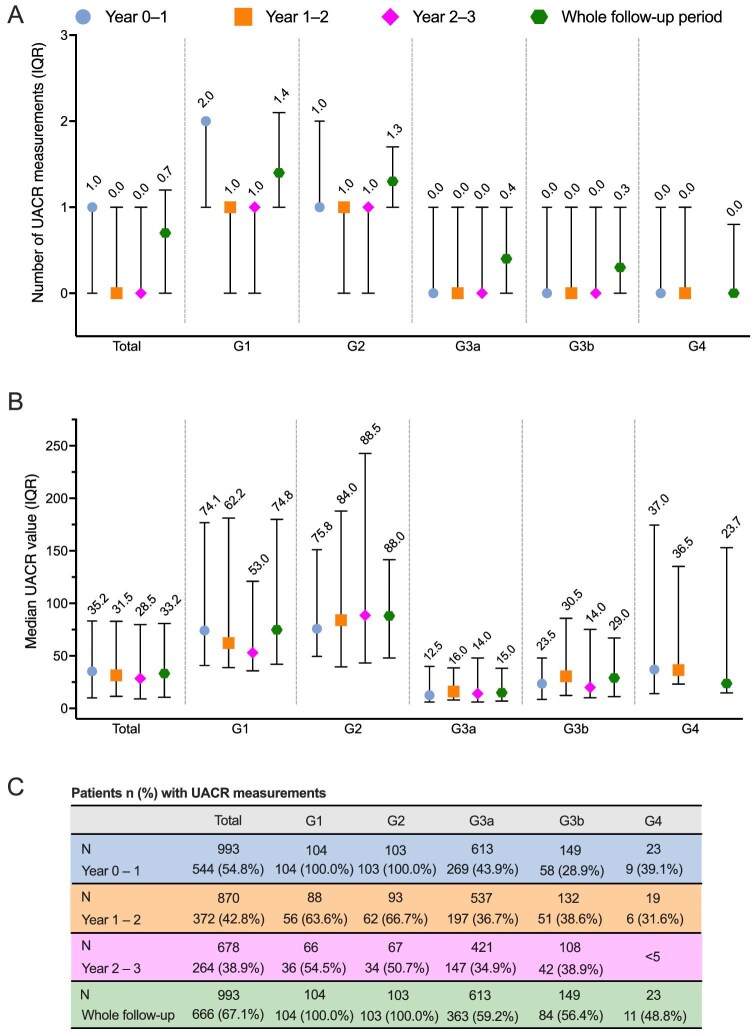
Median number and values of UACR measurements during 3 years of follow-up in the study population, total and by CKD G-stage at index. (**A**) Median number of UACR measurements, (**B**) median UACR values, and (**C**) number and fraction of patients with UACR measurements. All measurements taken during a given year were included in the calculation of the number and values of UACR values for that year. For the whole follow-up period analyses, all measurements during the follow-up were included and the annual number of measurements was calculated by dividing by the length of a follow-up.

During the first and second year of follow-up, the median number of recorded eGFR measurements was 2.0 per patient per year (Fig. 4). During the first year, the median number of measurements was higher for patients in stages G3b and G4 than in stages G1–G3a, while such a difference was not observed in the subsequent years. The number of UACR measurements was lower than the number of eGFR measurements during the first year of follow-up (median 1.0, IQR 0.0–1.0) and remained lower also in subsequent years (median per year 0.0, IQR 0.0–1.0) (Fig. 5).

### GP contacts and referrals to nephrologists during follow-up

The median number of GP contacts (in-person, online, and phone consultations) for CKD patients was 13.5 contacts/year (IQR 8.6–22.0, Table 3), and highest in stage G4 patients (17.9; IQR 9.0–24.6). A total of 27 (2.7%) patients were referred to a nephrologist during follow-up, with the highest proportion observed among stage G4 patients where 6 out of 23 (26.1%) patients were referred to a nephrologist during follow-up. The median follow-up time until referral was 2.3 years (IQR 1.6–3.0). The median values for measurements taken closest but prior to the time of referral were 30 mL/min/1.73 m^2^ (IQR 24.5–39.5) for eGFR and 98 mg/g (IQR 25.5–803.5) for UACR (Table 3).

**Table 3:** Follow-up information, GP contacts and referrals to a nephrologist.

	Stratification by CKD G-stage at index					
	Total (*N* = 993, 100.0%)^a^	G1 (*n* = 104, 10.5%)	G2 (*n* = 103, 10.4%)	G3a (*n* = 613, 61.8%)	G3b (*n* = 149, 15.0%)	G4 (*n* = 23, 2.3%)
Follow-up time (years)						
Median (Q1, Q3)	2.6 (1.7, 3.3)	2.4 (1.6, 3.0)	2.3 (1.7, 2.8)	2.6 (1.7, 3.4)	2.6 (1.7, 3.5)	2.7 (1.5, 3.2)
Censoring events, *n* (%)						
Death	55 (5.5)	0 (0.0)	<5	38 (6.2)	13 (8.7)	<5
Change of GP or emigration	23 (2.3)	<5	<5	15 (2.4)	<5	<5
Patients referred to a nephrologist	27 (2.7)	<5	<5	7 (1.1)	11 (7.4)	6 (26.1)
End of data collection period	888 (89.4)	100 (96.2)	100 (97.1)	553 (90.2)	121 (81.2)	13 (56.5)
GP contacts						
Number per patients per year during the entire follow-up						
median (Q1, Q3)	13.5 (8.6, 22.0)	13.3 (8.3, 22.6)	14.0 (9.0, 21.8)	13.2 (8.7, 21.0)	13.8 (8.8, 25.1)	17.9 (9.0, 24.6)
					**Total (*N* = 993, 100.0%)**	
Referral to a nephrologist^b^						
Patients referred, *n* (%)					27 (2.7)	
Time to referral in years since inclusion date, median (Q1, Q3)					2.3 (1.6, 3.0)	
Closest eGFR value prior to the referral						
Referred patients with measurement, *n* (%)					27 (2.7)	
Closest eGFR value prior to the referral (mL/min/1.73 m^b^), median (Q1, Q3)					30.0 (24.5, 39.5)	
Median time from measurement to referral in days (Q1, Q3)					18 (4, 175)	
Closest UACR value prior to the referral						
Referred patients with measurement, *n* (%)					19 (1.9)	
Closest UACR value to the referral (mg/g), median (Q1, Q3)					98.0 (25.5, 803.5)	

a Patients with unknown CKD stage are not reported separately but included in the ‘total’ column due to low number of observations (<5).

b There were not enough patients to present results regarding referrals stratified by G-stage.

Q1, first quartile; Q3, third quartile.

### eGFR decline during follow-up

Individual eGFR values varied between and within patients during the follow-up (Fig. 4). Based on all available eGFR measurements from index up to 3 years of follow-up, a patient experienced a median decline of –0.4 mL/min/1.73 m^2^ (IQR –3.0 to 2.0) in eGFR during that time period (Table 4). A total of 132 patients (15%) had a median decline in eGFR of >5 mL/min/1.73 m^2^ during the 3-year follow-up. Changes in CKD G-stage during follow-up are depicted in Supplementary data, Fig. S1.

**Table 4:** Change in eGFR by linear fit analysis and number (%) of patients with a decline in eGFR >5 mL/min/1.73 m^2^ analyzed in the whole cohort of patients with at least two eGFR measurements available and by CKD G-stage at index.

	Stratification by CKD G-stage at index (*n*, patients)					
Slope of eGFR change	Total (*N* = 993)^1^	G1 (*n* = 104)	G2 (*n* = 103)	G3a (*n* = 613)	G3b (*n* = 149)	G4 (*n* = 23)
Patients with at least two eGFR measurements available, *n* (%)	892 (89.8)	85 (81.7)	93 (90.3)	552 (90.0)	140 (94.0)	22 (95.7)
Median (Q1, Q3), mL/min/1.73 m^2^ change in eGFR during 3 years of follow-up	–0.4 (–3.0, 2.0)	–0.0 (–1.0, 0.0)	–1.2 (–4.4, 2.1)	–0.5 (–3.1, 2.4)	–0.7 (–2.9, 1.6)	0.1 (–1.6, 2.6)
Patients with eGFR decline of >5 mL/min/1.73 m^2^ during three years of follow-up, *n* (%)	132 (14.8)	10 (11.8)	21 (22.6)	84 (15.2)	15 (10.7)	<5

All available eGFR measurements from index up to 3 years of follow-up are taken into account in this analysis.

## DISCUSSION

In this nationwide cohort study of CKD patients in Danish primary care, 91% of all identified potential CKD patients had a second laboratory measurement performed ≥90 days after the initial test, and 75% had confirmed CKD based on a second test confirming declining kidney function ≥90 days after the initial test, as suggested by KDIGO guidelines [4]. This indicates that the likelihood of CKD following a single, abnormal eGFR or UACR measurement in primary care is high. However, the median time from patients included based on a single impaired kidney function measure to the second confirmatory test was 11 months. Thus, to allow for earlier initiation of relevant therapy, there may be a need for increased awareness of repeated testing after 3 months.

Although both eGFR and UACR tests are crucial components of CKD identification and the tests are readily available, testing for albuminuria remains insufficiently utilized in primary care. Almost half of the patients did not have a UACR measurement at the time of confirmed CKD identification. The underutilization of UACR is consistent with other studies [22–25], although UACR was more frequently recorded in this study. Possible explanations could include variations in selected patient populations based on applied algorithms to define CKD [26] and/or variations across healthcare settings and patients’ access to being tested. Borg *et al*. reported that only 3%–7% of a CKD study population (defined by two or more eGFR measurements) followed in primary care in the Copenhagen area of Denmark between 2011 and 2016 had an albuminuria assessment [18]. Moreover, most of the patients were at CKD stage G1 or G2 and the baseline prevalence of diabetes was 8%–10% as compared with 35% diabetics in our study. The different calendar periods for the studies may also have impacted the findings, as the importance of CKD including proteinuria and the context of comorbidities have likely gained more attention over time. Another and more recent Danish study examining eGFR from both primary and secondary care showed that one in three patients did not have UACR assessed in the year prior to incident CKD stage G3 [24]. This may indicate that GPs prescribe UACR more frequently than in the hospital setting, although this may also highly depend on healthcare providers purpose and incentives for UACR testing.

During follow-up, patients had regular monitoring of eGFR with one to two measurements per year, while the equivalent frequency of UACR tests remained lower (0–1 tests/year) during follow-up. In population studies, albuminuria with eGFR >60 mL/min/1.73 m^2^ defines CKD condition in more than half of CKD patients [27]. Also, albuminuria is an important risk factor for CKD progression, CVD and death, as well as an important determinant for the selection of treatment in both diabetic and non-diabetic CKD patients [4, 24, 28]. Thus, more frequent use of early albuminuria screening can improve the identification of CKD patients and provide an opportunity for intervention while eGFR is still well preserved [28].

Comorbidities were common among CKD patients and nearly 75% of the patients had hypertension [18], which is as expected considering that 73% of patients were ≥70 years old. As CVD is the most important cause of death in CKD patients, these findings underline the importance of treatment and follow-up [29, 30].

Two out of three CKD patients did not have diabetes, similar to findings in a recent multi-country study [31]. Notably, diabetes was more frequently observed among patients in CKD stages G1 and G2, suggesting that albuminuria without significant decline in eGFR may be a more common manifestation of CKD among patients with diabetes and/or that albuminuria is more frequently tested among diabetics. The latter highlights the importance of CKD screening, including albuminuria testing, among other risk patients. This may pose a challenge, as such patients might not undergo guideline-directed, regular screening for kidney disease to the same extent as patients with diabetes [32].

Treatment implementation to attenuate kidney disease progression after CKD diagnosis is known to be slow [31, 33]. However, this study showed generally positive findings of RAASi and statins being actively prescribed to most patients and remained stable during the study period. RAASi utilization among stage G3-4 patients in this study was greater when compared with the previous Danish primary care study [18] reporting that 38% of CKD stage G3–4 patients were treated with RAASi. In contrast, SGLT-2i were only prescribed to a minority of patients. SGLT-2i prescriptions increased significantly during the study period; however, this drug remained underused, as recently also observed elsewhere [7]. Only a few CKD patients without diabetes had SGLT-2i at the end of follow-up, which may, at least partly, be explained by SGLT-2i becoming available for the treatment of non-diabetic CKD only during the last 1.5 years of the follow-up period [34].

Very few patients were referred to a nephrologist. The median eGFR at referral was consistent with the Danish recommendations for referral (eGFR of 30 mL/min/1.73 m^2^ unless otherwise earlier indicated) [35]. According to these recommendations, the 23 patients identified with stage G4 CKD at index should perhaps already have been referred to a nephrologist prior to inclusion (which could be due to lack of sufficient look-back data to fulfil this exclusion criterion) or have obtained a referral during the follow-up period after index, which only 6 in 23 (26%) of the stage G4 CKD patients did. The median eGFR value closest to referral for these six patients were 25 mL/min/1.73 m^2^ and the median time to referral was 1.9 years (data not shown). Supplementary data, Fig. S1 indicates that 30% of patients with CKD stage G4 at index had in improvement in eGFR during 3 years of follow-up, which moved them to stage G3b. This could be a valid reason for not referring such patient. However, these referral patterns are based on small numbers, especially within the subgroup of stage G4 CKD patients, and should be interpreted with caution when inferring lack of guideline adherence for referral practice.

Among all confirmed CKD patients, the median UACR at referral was in the low-to-moderate albuminuria range, suggesting that albuminuria was not the main cause for referral. Consistent with this, fewer patients had UACR measured prior to referral compared with eGFR measurements. Overall, the large CKD patient population with frequent annual GP contacts and relatively few patients advancing to secondary care by referrals, emphasize the large responsibility of primary care to manage CKD and allocate resources to its management.

The overall rate of eGFR decline was relatively slow during follow-up, which may also be explained by the elderly patient population; however, 15% of patients had a median eGFR decline >5 mL/min/1.73 m^2^ within 3 years. Notably, 23% of patients in CKD stage G2 had such a decline, illustrating the importance of early CKD detection to prevent progression [11, 24]. Furthermore, different means of assessing the progression risk, such as risk calculators, could be included in routine clinical care [36–38].

This study utilized EHRs from primary care clinics representing all areas of Denmark, enabling analysis of unique data yet unavailable from Danish registers. The random sampling of patients suggests that patients were representative of the larger primary care CKD population and reduced potential selection bias. Identification of the study population by eGFR and UACR measurements allowed for the inclusion of patients not having been diagnosed as CKD patients. This is a major strength of the study as CKD is generally underdiagnosed. Furthermore, eGFR decline was estimated using all available eGFR measurements during follow-up, reducing the risk of misinterpretation due to a single, incorrect measurement and/or acute kidney injury. However, some limitations apply. The quality assessment nature of the study's legal framework restricted the data collection period to a maximum of 5 years, which may have resulted in a lack of important historical data. Medication data was derived from prescriptions and thus might not fully reflect the actual medication adherence. Despite the a priori exclusion of patients that had already been referred to nephrologist prior to inclusion, the study population most likely did not only include incident but also prevalent CKD cases as there was no wash-out period prior to the first assessment for CKD. This also implies that the estimated time between the first and a confirmatory test may be biased as some patients may have already been identified as having prevalent CKD. As data from this primary care setting were not linked with any other health data sources, we did not explore the impact of being identified with CKD based on a single or several confirmatory tests and test intervals on patient's prognosis and clinical outcomes. Furthermore, we did not collect data assessing the reason for GP contacts and (lack of) treatment initiation and/or referral. Thus, we cannot make definite conclusions on the cause of GP contacts and clinical decision making for rationalization of treatment and/or referral strategies among the included CKD patients. The accuracy of CKD progression with eGFR decline over time should be interpreted with caution due to limited number of eGFR measurements (median 2.3) during follow-up. Lastly, some bias may have been introduced in the representation of primary care clinics that chose to participate in this study, although the 134 clinics broadly represent all geographical areas of Denmark.

In conclusion, this study provides valuable information on CKD management in Danish primary care. While the overall findings were positive and showed high quality of care among CKD patients in primary care, such as frequency of eGFR testing and prescriptions of RAASi and statins, some areas could be optimized for improved care. This includes early CKD identification with more frequent UACR tests and increased adaptions to new treatments, such as SGLT-2i, to reduce the risk of CKD progression and the associated morbidity and mortality.

### Legal framework

The study was conducted as a non-interventional quality-assurance project in Denmark. As such, it does not require specific legal approvals or informed consent from the included patients.

## Supplementary Material

sfae393_Supplemental_File

## Data Availability

Data were collected and analyzed as anonymized data, as part of quality assurance purpose from GP healthcare records. Only the participating GPs have access to the individual-level data which cannot be shared due to ethical/privacy reasons. All analyzed data are incorporated into the article and its online supplementary material.
