# Correction to: The management of chronic kidney disease in primary care in Denmark: patient characteristics, treatment, follow-up, progression and referral

**DOI:** 10.1093/ckj/sfaf177

**Published:** 2025-07-01

This is a correction to: Henrik Birn, Karl Emil Nelveg-Kristensen, Line Elmerdahl Frederiksen, Stefan Christensen, Juha Mehtälä, Sarah Smith, Michael Bruun, Ulrik Bodholdt, The management of chronic kidney disease in primary care in Denmark: patient characteristics, treatment, follow-up, progression and referral, *Clinical Kidney Journal*, Volume 18, Issue 2, February 2025, sfae393, https://doi.org/10.1093/ckj/sfae393

The graphical abstract was inadvertently omitted from this paper on first publication. The graphical abstract has now been added.

