# Supplementary material for: The management of chronic kidney disease in primary care in Denmark: patient characteristics, treatment, follow-up, progression and referral
Source: Clin Kidney J. 2024 Nov 30;18(2):sfae393. doi: 10.1093/ckj/sfae393 (PMC11788563; doi:10.1093/ckj/sfae393)
Supplement: sfae393_Supplemental_File [file sfae393_supplemental_file.docx]

Supplementary Information

**Supplementary Table 1.** Definitions of chronic kidney disease (CKD) G-staging.

| **CKD Stage** | **Definition** |
| --- | --- |
| G1 | UACR >30mg/g at inclusion and at first available UACR test ≥90 days since inclusion, and eGFR ≥90ml/min/1.73m^2^ at first available test any time after inclusion |
| G2 | UACR >30mg/g at inclusion and at first available UACR test ≥90 days since inclusion, and eGFR 60-90ml/min/1.73m^2^ at first available test any time after inclusion. |
| G3a | eGFR <60ml/min/1.73m^2^ at inclusion and eGFR 45-59 ml/min/1.73m^2^ at first available test ≥90 days since inclusion.  OR  UACR >30mg/g at inclusion and at first available UACR test ≥90 days since inclusion, and eGFR 45-59 ml/min/1.73m^2^ at first available test any time after inclusion |
| G3b | eGFR <60ml/min/1.73m^2^ at inclusion and eGFR 30-44 ml/min/1.73m^2^ at first available test ≥90 days since inclusion.  OR  UACR >30mg/g at inclusion and at first available UACR test ≥90 days since inclusion, and eGFR 30-44 ml/min/1.73m^2^ at first available test any time after inclusion |
| G4 | eGFR <60ml/min/1.73m^2^ at inclusion and eGFR 15-29 ml/min/1.73m^2^ at first available test ≥90 days since inclusion.  OR  UACR >30mg/g at inclusion and at first available UACR test ≥90 days since inclusion, and eGFR 15-29 ml/min/1.73m^2^ at first available test any time after inclusion. |
| G5 | eGFR <60ml/min/1.73m^2^ at inclusion and eGFR <15 ml/min/1.73m^2^ at first available test ≥90 days since inclusion.  OR  UACR >30mg/g at inclusion and at first available UACR test ≥90 days since inclusion, and eGFR <15 ml/min/1.73m^2^ at first available test any time after inclusion. |
| Unknown | UACR >30mg/g at inclusion and at first available UACR test ≥90 days since inclusion, and eGFR nor measured at any point after inclusion. |

**Abbreviations:** CKD; chronic kidney disease, eGFR; estimated glomerular filtration rate, UACR; urine albumin-creatinine ratio

**Supplementary Table 2.** Patient and clinical characteristics and proportion of patients in the study population with an active prescription of different medication groups at the inclusion and the end of follow-up, stratified by diabetes status at the inclusion date.

|  | Total (N=993) | No diabetes (n=642) | Diabetes (n=351) |
| --- | --- | --- | --- |
| **Follow-up time (years)** |  |  |  |
| Median (Q1, Q3) | 2.6 (1.7, 3.3) | 2.5 (1.7, 3.3) | 2.6 (1.8, 3.5) |
| **Censoring events, n (%)** |  |  |  |
| Patients referred to a nephrologist | 27 (2.7%) | 15 (2.3%) | 12 (3.4%) |
| End of data collection period | 578 (90.0%) | 310 (88.3%) | 888 (89.4%) |
| Death | 55 (5.5%) | 33 (5.1%) | 22 (6.3%) |
| Change of GP or emigration | 23 (2.3%) | 16 (2.5%) | 7 (2.0%) |
| **GP consultations, n (%)** |  |  |  |
| Number per patients per year over the whole follow-up, median (Q1, Q3) | 13.5 (8.6, 22.0) | 12.1 (8.3, 19.1) | 17.2 (10.3, 24.0) |
| **Sociodemographic characteristics** |  |  |  |
| **Gender, n (%)** |  |  |  |
| Female | 534 (53.8%) | 363 (56.5%) | 171 (48.7%) |
| Male | 459 (46.2%) | 279 (43.5%) | 180 (51.3%) |
| **Age group, n (%)** |  |  |  |
| <59 years | 101 (10.2%) | 37 (5.7%) | 64 (18.2%) |
| 60-69 years | 169 (17.0%) | 98 (15.3%) | 71 (20.2%) |
| 70-79 years | 393 (39.6%) | 264 (41.1%) | 129 (36.8%) |
| 80 years or over | 330 (33.2%) | 243 (37.9%) | 87 (24.8%) |
| **Smoking, n (%)** |  |  |  |
| Current smoker | 148 (14.9%) | 88 (13.7%) | 60 (17.1%) |
| Former smoker | 258 (26.0%) | 157 (24.5%) | 101 (28.8%) |
| Never smoked | 461 (46.4%) | 305 (47.5%) | 156 (44.4%) |
| Missing | 126 (12.7%) | 92 (14.3%) | 34 (9.7%) |
| **CKD-related information** |  |  |  |
| **UACR at index (measured within 7 days after index date), n (%)** |  |  |  |
| Available | 520 (52.4%) | 295 (46.0%) | 225 (64.1%) |
| **Other previous renal-related diseases or surgery, n (%)** |  |  |  |
| No | 930 (93.7%) | 601 (93.6%) | 329 (93.7%) |
| Acute renal failure | 7 (0.7%) | NA | NA |
| Renal cancer | 9 (0.9%) | NA | NA |
| Surgery on kidney or bladder | 21 (2.1%) | 14 (2.2%) | 7 (2.0%) |
| Other | 33 (3.3%) | 21 (3.3%) | 12 (3.4%) |
| **Comorbidities at time of inclusion** |  |  |  |
| **Cardiovascular disease at inclusion, n (%)** |  |  |  |
| Hypertension | 734 (73.9%) | 464 (72.3%) | 270 (76.9%) |
| Ischemic heart disease | 140 (14.1%) | 86 (13.4%) | 54 (15.4%) |
| Heart failure | 85 (8.6%) | 48 (7.5%) | 37 (10.5%) |
| Stroke | 72 (7.3%) | 41 (6.4%) | 31 (8.8%) |
| Peripheral vascular disease | 44 (4.4%) | 29 (4.5%) | 15 (4.3%) |
| Other cardiovascular diseases | 410 (41.3%) | 241 (37.5%) | 169 (48.1%) |
| **Other cardiovascular diseases at inclusion, n (%)** |  |  |  |
| Diabetic vascular complications | 44 (4.4%) | 0 (0.0%) | 44 (4.4%) |
| Peripheral artery diseases | 144 (14.5%) | 86 (13.4%) | 58 (16.5%) |
| Cardiac valve disorders | 71 (7.2%) | 48 (7.5%) | 23 (6.6%) |
| Atrial fibrillation | 176 (17.7%) | 111 (17.3%) | 65 (18.5%) |
| Cerebrovascular disease excluding stroke, other occlusions of cerebral arteries and transient cerebral ischemic attack | 37 (3.7%) | 23 (3.6%) | 14 (4.0%) |
| Cardiac arrest | <5 | <5 | 0 (0.0%) |
| **Urinary tract infection at inclusion, n (%)** |  |  |  |
| Yes | 43 (4.3%) | 24 (3.7%) | 19 (5.4%) |
| **Clinical characteristics at inclusion (± 4 weeks)** |  |  |  |
| Hemoglobin measurement available | 801 (80.7%) | 534 (83.2%) | 267 (76.1%) |
| Hemoglobin (mmol/l), median (Q1, Q3) | 8.4 (7.9, 9.1) | 8.4 (7.8, 9.0) | 8.6 (7.9, 9.3) |
| HbA1c measurement available | 804 | 479 | 325 |
| HbA1c (mmol/mol), median (Q1, Q3) | 41.0 (37.0, 48.0) | 38.0 (36.0, 41.0) | 51.0 (45.0, 62.0) |
| LDL measurement available | 717 | 458 | 259 |
| LDL (mmol/L), median (Q1, Q3) | 2.3 (1.7, 3.1) | 2.5 (1.9, 3.3) | 1.9 (1.4, 2.4) |
| Systolic BP measurement available | 701 | 439 | 262 |
| Systolic BP (mmHg), median (Q1, Q3) | 136.0 (129.0, 145.0) | 136.0 (129.0, 145.5) | 135.0 (129.0, 145.0) |
| Diastolic BP measurement available | 699 | 438 | 261 |
| Diastolic BP (mmHg), median (Q1, Q3) | 79.0 (71.5, 85.0) | 79.0 (72.0, 85.0) | 79.0 (70.0, 85.0) |
| **Number and proportion of patients with abnormal measurements** |  |  |  |
| Low Hemoglobin (mmol/l) | 155 (19.4%) | 98 (18.4%) | 57 (21.3%) |
| Elevated HbA1c (mmol/mol) | 198 (24.6%) | NA | NA |
| Elevated LDL (mmol/L) | 180 (25.1%) | 146 (31.9%) | 34 (13.1%) |
| High BP (mmHg) | 474 (67.6%) | 299 (68.1%) | 175 (66.8%) |
| **Active prescription of medication group, n (%)** |  |  |  |
| **RAASi** |  |  |  |
| At the inclusion date | 666 (67.1%) | 407 (63.4%) | 259 (73.8%) |
| End of follow-up | 729 (73.4%) | 449 (69.9%) | 280 (79.8%) |
| **SGLT-2i** |  |  |  |
| At the inclusion date | 49 (4.9%) | NA | NA |
| End of follow-up | 151 (15.2%) | 19 (3.0%) | 132 (37.6%) |
| **Statins** |  |  |  |
| At the inclusion date | 506 (51.0%) | 282 (43.9%) | 224 (63.8%) |
| End of follow-up | 569 (57.3%) | 331 (51.6%) | 238 (67.8%) |
| **NSAIDs** |  |  |  |
| At the inclusion date | 46 (4.6%) | 28 (4.4%) | 18 (5.1%) |
| End of follow-up | 43 (4.3%) | 25 (3.9%) | 18 (5.1%) |
| **MRA** |  |  |  |
| At the inclusion date | 87 (8.8%) | 50 (7.8%) | 37 (10.5%) |
| End of follow-up | 123 (12.4%) | 73 (11.4%) | 50 (14.2%) |
| **Annual eGFR measurements** |  |  |  |
| **Median number of measurements (Q1, Q3)** |  |  |  |
| Year 0 – 1 | 2.0 (1.0, 4.0) | 2.0 (1.0, 3.0) | 3.0 (2.0, 4.0) |
| Year 1 – 2 | 2.0 (1.0, 3.0) | 1.0 (1.0, 2.0) | 2.0 (1.0, 4.0) |
| Year 2 – 3 | 1.0 (1.0, 2.0) | 1.0 (0.0, 2.0) | 2.0 (1.0, 3.0) |
| Whole follow-up | 2.3 (1.5, 3.6) | 2.0 (1.3, 3.1) | 2.9 (1.9, 4.0) |
| **Median value (ml/min/1.73m2), (Q1, Q3)** |  |  |  |
| Year 0 – 1 | 54.0 (47.3, 60.0) | 53.8 (47.0, 58.0) | 57.0 (48.0, 78.3) |
| Year 1 – 2 | 53.0 (44.8, 62.7) | 52.3 (44.0, 60.0) | 56.6 (45.5, 74.8) |
| Year 2 – 3 | 52.0 (43.0, 62.0) | 51.0 (43.0, 58.5) | 53.8 (42.5, 71.0) |
| Whole follow-up | 53.8 (46.4, 60.6) | 53.0 (46.4, 58.0) | 56.2 (46.4, 77.8) |
| **Patients with eGFR measurement (n, %)** |  |  |  |
| Year 0 – 1 | 984 (99.1%) | 636 (99.1%) | 348 (99.1%) |
| Year 1 – 2 | 688 (79.1%) | 424 (75.6%) | 264 (85.4%) |
| Year 2 – 3 | 510 (75.2%) | 305 (70.9%) | 205 (82.7%) |
| Whole follow-up | 988 (99.5%) | 639 (99.5%) | 349 (99.4%) |
| **Annual UACR measurements** |  |  |  |
| **Median number of measurements (Q1, Q3)** |  |  |  |
| Year 0 – 1 | 1.0 (0.0, 1.0) | 0.0 (0.0, 1.0) | 1.0 (0.0, 2.0) |
| Year 1 –2 | 0.0 (0.0, 1.0) | 0.0 (0.0, 1.0) | 1.0 (0.0, 1.0) |
| Year 2 – 3 | 0.0 (0.0, 1.0) | 0.0 (0.0, 1.0) | 1.0 (0.0, 1.0) |
| Whole follow-up | 0.7 (0.0, 1.2) | 0.4 (0.0, 1.0) | 1.0 (0.4, 1.5) |
| **Median value (mg/g) (Q1, Q3)** |  |  |  |
| Year 0 – 1 | 35.2 (10.0, 83.2) | 24.4 (8.0, 62.2) | 49.0 (26.4, 125.0) |
| Year 1 – 2 | 31.5 (11.5, 82.9) | 20.0 (9.0, 65.0) | 42.0 (16.0, 114.0) |
| Year 2 – 3 | 28.5 (9.0, 79.7) | 18.0 (6.0, 49.0) | 44.0 (14.0, 98.5) |
| Whole follow-up | 33.2 (10.5, 80.9) | 21.8 (8.0, 59.0) | 50.1 (24.0, 125.5) |
| **Patients with UACR measurement (n, %)** |  |  |  |
| Year 0 – 1 | 544 (54.8%) | 296 (46.1%) | 248 (70.7%) |
| Year 1 – 2 | 372 (42.8%) | 191 (34.0%) | 181 (58.6%) |
| Year 2 – 3 | 264 (38.9%) | 127 (29.5%) | 137 (55.2%) |
| Whole follow-up | 666 (67.1%) | 384 (59.8%) | 282 (80.3%) |

**Abbreviations:** BP, blood pressure; CKD, chronic kidney disease; eGFR, estimated glomerular filtration rate; GP, General practitioner; HbA1c, Hemoglobin A1c; LDL, low-density lipoprotein; MRA, Mineralocorticoid receptor antagonist; NA, not available (one of two subgroups included <5 patients and thus they are masked); NSAID, Non-steroidal anti-inflammatory drug; Q1, first quartile; Q3, third quartile; RAASi, Renin-angiotensin-aldosterone system inhibitor; SGLT-2i, Sodium-glucose cotransporter 2 inhibitor; UACR, urine albumin-creatinine ratio.

**Supplementary Table 3.** Baseline sociodemographic and clinical characteristics of confirmed CKD patients and among the 102 patients with only a single kidney function measurement available or no second measurement that fulfilled the eligibility measurement to confirm CKD

|  | **Confirmed CKD patients (N=993)** | **Patients with a single eligible eGFR or UACR test* (N=102)** |
| --- | --- | --- |
| **Sociodemographic characteristics** | | |
| **Gender, n (%)** |  |  |
| Female | 534 (53.8%) | 49 (48.0%) |
| Male | 459 (46.2%) | 53 (52.0%) |
| **Age group, n (%)** |  |  |
| <59 years | 101 (10.2%) | 33 (32.3%) |
| 60-69 years | 169 (17.0%) | 19 (18.6%) |
| 70-79 years | 393 (39.6%) | 33 (32.4%) |
| 80 years or over | 330 (33.2%) | 19 (18.6%) |
| **Smoking, n (%)** |  |  |
| Current smoker | 148 (14.9%) | 20 (19.6%) |
| Former smoker | 258 (26.0%) | 16 (15.7%) |
| Never smoked | 461 (46.4%) | 32 (31.4%) |
| Missing | 126 (12.7%) | 34 (33.3%) |
| **CKD-related information** | | |
| **UACR at index (measured within 7 days after index date), n (%)** | | |
| A1 (<30mg/g) | 207 (39.8%) | 1-5 |
| A2 (30–300mg/g) | 273 (52.5%) | 46 (83.6%) |
| A3 (>300mg/g) | 40 (7.7%) | 1-5 |
| Missing | 473 (47.6%) | 47 (46.1%) |
| **Cause of CKD, n (%)** |  |  |
| Diabetic kidney disease | 223 (22.5%) | 21 (20.6%) |
| Hypertensive nephropathy | 285 (28.7%) | 17 (16.7%) |
| Atherosclerosis of renal artery | 41 (4.1%) | <5 |
| Obstructive nephropathy | 32 (3.2%) | 0 (0.0%) |
| Unknown^2^ | 504 (50.8%) | 68 (66.7%) |
| **Other previous renal-related diseases or operations, n (%)** | | |
| No^2^ | 930 (93.7%) | 97 (95.1%) |
| Acute renal failure | 7 (0.7%) | <5 |
| Renal cancer | 9 (0.9%) | 0 (0.0%) |
| Surgery on kidney or bladder | 21 (2.1%) | <5 |
| Other | 33 (3.3%) | <5 |
| **Comorbidities** | | |
| **Diabetes at inclusion, n (%)** | | |
| Type 1 diabetes | 15 (1.5%) | <5 |
| Type 2 diabetes | 336 (33.8%) | 24 (23.5%) |
| **Cardiovascular disease at inclusion, n (%)** | | |
| Hypertension | 734 (73.9%) | 49 (48.0%) |
| Ischemic heart disease | 140 (14.1%) | 11 (10.8%) |
| Heart failure | 85 (8.6%) | 7 (6.9%) |
| Stroke | 72 (7.3%) | <5 |
| Peripheral vascular disease | 44 (4.4%) | 9 (8.8%) |
| Other cardiovascular diseases | 410 (41.3%) | 25 (24.5%) |
| **Urinary tract infection at inclusion, n (%)** | | |
| Yes | 43 (4.3%) | 5 (4.9%) |
| **Laboratory measurements at inclusion (+/- 4 weeks)** | | |
| Hemoglobin (mmol/l), median | 8.5 | 8.6 |
| HbA1c (mmol/mol), median | 41.0 | 38.0 |
| LDL (mmol/L), median | 2.2 | 2.4 |
| Systolic BP (mmHg), median | 136.0 | 139.0 |
| Diastolic BP (mmHg), median | 79.0 | 80.0 |
| **Prescribed medication at inclusion** | | |
| RAASi, % | 67.1% | 47.1% |
| SGLT-2i, % | 4.9% | 4.9% |
| Statins, % | 51.0% | 35.3% |
| NSAIDs, % | 4.6% | <5 |
| MRAs, % | 8.8% | 5.9% |
| **Annual GP consultations** | | |
| Median (Q1, Q3) | 13.5 (8.6, 22.0) | 10.0 (5.0, 21.9) |

* Patients with a single measurement over whole study period or patients that have eGFR at index with no second eGFR measurement taken ever or patients with UACR at index with no second UACR measurement taken ever.


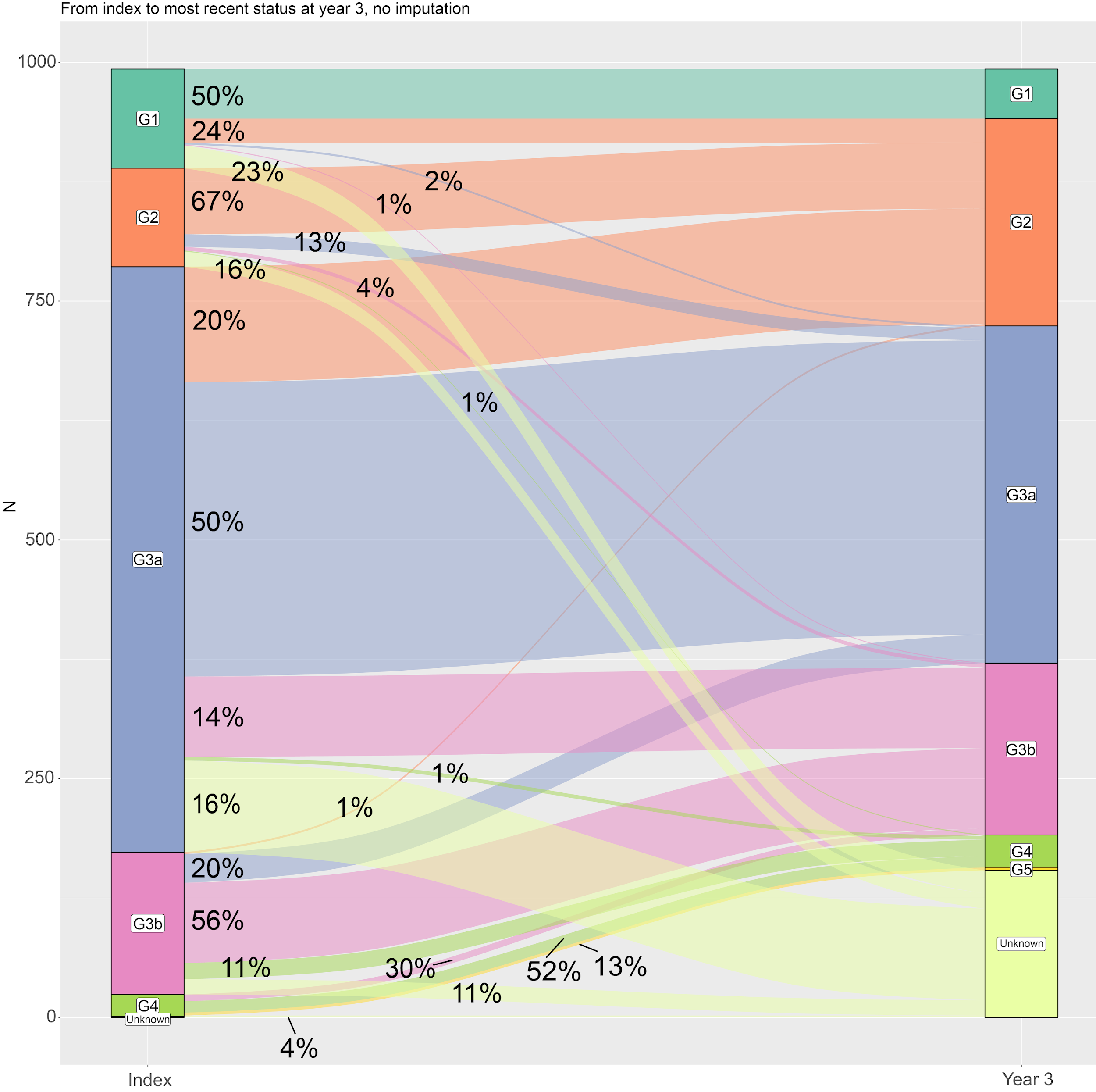


**Supplementary Figure 1. Change in CKD stage during 3 years of follow-up.** A cross-sectional view on the proportion of patients in different CKD stages at the index and a maximum of three years end of follow-up (based on the last measurement available up to 3 years).

*Unknown*: a patient does not have a second eGFR value available after the confirmatory measurement due to any reason (no measurement, loss to follow-up etc.). The colors of the stacked bars indicate the different CKD stages. The lines between the stacked bars and associated % indicate the movement and proportion of patients between CKD stages.
